# Author Correction: Spread of pathological tau proteins through communicating neurons in human Alzheimer’s disease

**DOI:** 10.1038/s41467-021-25193-3

**Published:** 2021-08-05

**Authors:** Jacob W. Vogel, Yasser Iturria-Medina, Olof T. Strandberg, Ruben Smith, Elizabeth Levitis, Alan C. Evans, Oskar Hansson, Alzheimer’s Disease Neuroimaging Initiative, Michael Weiner, Paul Aisen, Ronald Petersen, Clifford R. Jack, William Jagust, John Q. Trojanowki, Arthur W. Toga, Laurel Beckett, Robert C. Green, Andrew J. Saykin, John Morris, Leslie M. Shaw, Enchi Liu, Tom Montine, Ronald G. Thomas, Michael Donohue, Sarah Walter, Devon Gessert, Tamie Sather, Gus Jiminez, Danielle Harvey, Michael Donohue, Matthew Bernstein, Nick Fox, Paul Thompson, Norbert Schuff, Charles DeCArli, Bret Borowski, Jeff Gunter, Matt Senjem, Prashanthi Vemuri, David Jones, Kejal Kantarci, Chad Ward, Robert A. Koeppe, Norm Foster, Eric M. Reiman, Kewei Chen, Chet Mathis, Susan Landau, Nigel J. Cairns, Erin Householder, Lisa Taylor Reinwald, Virginia Lee, Magdalena Korecka, Michal Figurski, Karen Crawford, Scott Neu, Tatiana M. Foroud, Steven Potkin, Li Shen, Faber Kelley, Sungeun Kim, Kwangsik Nho, Zaven Kachaturian, Richard Frank, Peter J. Snyder, Susan Molchan, Jeffrey Kaye, Joseph Quinn, Betty Lind, Raina Carter, Sara Dolen, Lon S. Schneider, Sonia Pawluczyk, Mauricio Beccera, Liberty Teodoro, Bryan M. Spann, James Brewer, Helen Vanderswag, Adam Fleisher, Judith L. Heidebrink, Joanne L. Lord, Ronald Petersen, Sara S. Mason, Colleen S. Albers, David Knopman, Kris Johnson, Rachelle S. Doody, Javier Villanueva Meyer, Munir Chowdhury, Susan Rountree, Mimi Dang, Yaakov Stern, Lawrence S. Honig, Karen L. Bell, Beau Ances, John C. Morris, Maria Carroll, Sue Leon, Erin Householder, Mark A. Mintun, Stacy Schneider, Angela OliverNG, Randall Griffith, David Clark, David Geldmacher, John Brockington, Erik Roberson, Hillel Grossman, Effie Mitsis, Leyla de Toledo-Morrell, Raj C. Shah, Ranjan Duara, Daniel Varon, Maria T. Greig, Peggy Roberts, Marilyn Albert, Chiadi Onyike, Daniel D’Agostino, Stephanie Kielb, James E. Galvin, Dana M. Pogorelec, Brittany Cerbone, Christina A. Michel, Henry Rusinek, Mony J. de Leon, Lidia Glodzik, Susan De Santi, P. Murali Doraiswamy, Jeffrey R. Petrella, Terence Z. Wong, Steven E. Arnold, Jason H. Karlawish, David Wolk, Charles D. Smith, Greg Jicha, Peter Hardy, Partha Sinha, Elizabeth Oates, Gary Conrad, Oscar L. Lopez, MaryAnn Oakley, Donna M. Simpson, Anton P. Porsteinsson, Bonnie S. Goldstein, Kim Martin, Kelly M. Makino, M. Saleem Ismail, Connie Brand, Ruth A. Mulnard, Gaby Thai, Catherine Mc Adams Ortiz, Kyle Womack, Dana Mathews, Mary Quiceno, Ramon Diaz Arrastia, Richard King, Myron Weiner, Kristen Martin Cook, Michael DeVous, Allan I. Levey, James J. Lah, Janet S. Cellar, Jeffrey M. Burns, Heather S. Anderson, Russell H. Swerdlow, Liana Apostolova, Kathleen Tingus, Ellen Woo, Daniel H. S. Silverman, Po H. Lu, George Bartzokis, Neill R. Graff Radford, Francine Parfitt, Tracy Kendall, Heather Johnson, Martin R. Farlow, Ann Marie Hake, Brandy R. Matthews, Scott Herring, Cynthia Hunt, Christopher H. van Dyck, Richard E. Carson, Martha G. MacAvoy, Howard Chertkow, Howard Bergman, Chris Hosein, Sandra Black, Bojana Stefanovic, Curtis Caldwell, Ging Yuek Robin Hsiung, Howard Feldman, Benita Mudge, Michele Assaly Past, Andrew Kertesz, John Rogers, Dick Trost, Charles Bernick, Donna Munic, Diana Kerwin, Marek Marsel Mesulam, Kristine Lipowski, Chuang Kuo Wu, Nancy Johnson, Carl Sadowsky, Walter Martinez, Teresa Villena, Raymond Scott Turner, Kathleen Johnson, Brigid Reynolds, Reisa A. Sperling, Keith A. Johnson, Gad Marshall, Meghan Frey, Jerome Yesavage, Joy L. Taylor, Barton Lane, Allyson Rosen, Jared Tinklenberg, Marwan N. Sabbagh, Christine M. Belden, Sandra A. Jacobson, Sherye A. Sirrel, Neil Kowall, Ronald Killiany, Andrew E. Budson, Alexander Norbash, Patricia Lynn Johnson, Thomas O. Obisesan, Saba Wolday, Joanne Allard, Alan Lerner, Paula Ogrocki, Leon Hudson, Evan Fletcher, Owen Carmichael, John Olichney, Charles DeCarli, Smita Kittur, Michael Borrie, T. Y. Lee, Rob Bartha, Sterling Johnson, Sanjay Asthana, Cynthia M. Carlsson, Steven G. Potkin, Adrian Preda, Dana Nguyen, Pierre Tariot, Adam Fleisher, Stephanie Reeder, Vernice Bates, Horacio Capote, Michelle Rainka, Douglas W. Scharre, Maria Kataki, Anahita Adeli, Earl A. Zimmerman, Dzintra Celmins, Alice D. Brown, Godfrey D. Pearlson, Karen Blank, Karen Anderson, Robert B. Santulli, Tamar J. Kitzmiller, Eben S. Schwartz, Kaycee M. SinkS, Jeff D. Williamson, Pradeep Garg, Franklin Watkins, Brian R. Ott, Henry Querfurth, Geoffrey Tremont, Stephen Salloway, Paul Malloy, Stephen Correia, Howard J. Rosen, Bruce L. Miller, Jacobo Mintzer, Kenneth Spicer, David Bachman, Elizabether Finger, Stephen Pasternak, Irina Rachinsky, John Rogers, Andrew Kertesz, Dick Drost, Nunzio Pomara, Raymundo Hernando, Antero Sarrael, Susan K. Schultz, Laura L. Boles Ponto, Hyungsub Shim, Karen Elizabeth Smith, Norman Relkin, Gloria Chaing, Lisa Raudin, Amanda Smith, Kristin Fargher, Balebail Ashok Raj, the Swedish BioFinder Study, Emelie Andersson, David Berron, Elin Byman, Tone Sundberg-Brorsson, Administrator, Emma Borland, Anna Callmer, Cecilia Dahl, Eske Gertje, Anna-Märta Gustavsson, Joanna Grzegorska, Sara Hall, Oskar Hansson, Philip Insel, Shorena Janelidze, Maurits Johansson, Helena Sletten, Jonas Jester-Broms, Elisabet Londos, Niklas Mattson, Lennart Minthon, Maria Nilsson, Rosita Nordkvist, Katarina Nägga, Camilla Orbjörn, Rik Ossenkoppele, Sebastian Palmqvist, Marie Persson, Alexander Santillo, Nicola Spotorno, Erik Stomrud, Håkan Toresson, Olof Strandberg, Michael Schöll, Ida Friberg, Per Johansson, Moa Wibom, Katarina Johansson, Emma Pettersson, Christin Karremo, Ruben Smith, Yulia Surova, Mattis Jalakas, Jimmy Lätt, Peter Mannfolk, Markus Nilsson, Freddy Ståhlberg, Pia Sundgren, Danielle van Westen, Ulf Andreasson, Kaj Blennow, Henrik Zetterberg, Lars-Olof Wahlund, Eric Westman, Joana Pereira, Jonas Jögi, Douglas Hägerström, Tomas Olsson, Per Wollmer

**Affiliations:** 1 Montreal Neurological Institute, McGill University, Montréal, QC Canada; 2 Clinical Memory Research Unit, Lund University, Lund, Sweden; 3 Memory Clinic, Skåne University Hospital, Lund, Sweden; 4 UC San Francisco, San Francisco, CA USA; 5 UC San Diego, San Diego, CA USA; 6 Mayo Clinic, Rochester, NY USA; 7 UC Berkeley, Berkeley, CA USA; 8 U Pennsylvania, Pennsylvania, CA USA; 9 USC, Los Angeles, CA USA; 10 UC Davis, Davis, CA USA; 11 Brigham and Women’s Hospital, Harvard Medical School, Boston, MA USA; 12 Indiana University, Bloomington, IN USA; 13 Washington University St. Louis, St. Louis, MO USA; 14 University of Pennsylvania, Philadelphia, PA USA; 15 Janssen Alzheimer Immunotherapy, South San Francisco, CA USA; 16 University of Washington, Seattle, WA USA; 17 University of London, London, UK; 18 USC School of Medicine, Los Angeles, CA USA; 19 UCSF MRI, San Francisco, CA USA; 20 University of Michigan, Ann Arbor, MI USA; 21 University of Utah, Salt Lake City, UT USA; 22 Banner Alzheimer’s Institute, Phoenix, AZ USA; 23 University of Pittsburgh, Pittsburgh, PA USA; 24 UPenn School of Medicine, Philadelphia, PA USA; 25 UC Irvine, Newport Beach, CA USA; 26 Khachaturian, Radebaugh & Associates, Inc and Alzheimer’s Association’s Ronald and Nancy Reagan’s Research Institute, Chicago, IL USA; 27 General Electric, Boston, MA USA; 28 Brown University, Providence, RI USA; 29 National Institute on Aging/National Institutes of Health, Bethesda, MD USA; 30 Oregon Health and Science University, Portland, OR USA; 31 University of Southern California, Los Angeles, CA USA; 32 University of California San Diego, San Diego, CA USA; 33 Baylor College of Medicine, Houston, TX USA; 34 Columbia University Medical Center, New York, NY USA; 35 Washington University, St. Louis, MO USA; 36 University of Alabama Birmingham, Birmingham, MO USA; 37 Mount Sinai School of Medicine, New York, NY USA; 38 Rush University Medical Center, Chicago, IL USA; 39 Wien Center, Vienna, Austria; 40 Johns Hopkins University, Baltimore, MD USA; 41 New York University, New York, NY USA; 42 Duke University Medical Center, Durham, NC USA; 43 University of Kentucky, city of Lexington, NC USA; 44 University of Rochester Medical Center, Rochester, NY USA; 45 University of California, Irvine, CA USA; 46 University of Texas Southwestern Medical School, Dallas, TX USA; 47 Emory University, Atlanta, GA USA; 48 University of Kansas, Medical Center, Lawrence, KS USA; 49 University of California, Los Angeles, CA USA; 50 Mayo Clinic, Jacksonville, FL USA; 51 Yale University School of Medicine, New Haven, CT USA; 52 McGill Univ., Montreal Jewish General Hospital, Montreal, WI USA; 53 Sunnybrook Health Sciences, Toronto, ON Canada; 54 U.B.C. Clinic for AD & Related Disorders, British Columbia, BC Canada; 55 Cognitive Neurology St. Joseph’s, Toronto, ON Canada; 56 Cleveland Clinic Lou Ruvo Center for Brain Health, Las Vegas, NV USA; 57 Northwestern University, Evanston, IL USA; 58 Premiere Research Inst Palm Beach Neurology, West Palm Beach, FL USA; 59 Georgetown University Medical Center, Washington, DC USA; 60 Brigham and Women’s Hospital, Boston, MA USA; 61 Stanford University, Santa Clara County, CA USA; 62 Banner Sun Health Research Institute, Sun City, AZ USA; 63 Boston University, Boston, MA USA; 64 Howard University, Washington, DC USA; 65 Case Western Reserve University, Cleveland, OH USA; 66 University of California, Davis Sacramento, CA USA; 67 Neurological Care of CNY, New York, NY USA; 68 Parkwood Hospital, Parkwood, CA USA; 69 University of Wisconsin, Madison, WI USA; 70 University of California, Irvine BIC, Irvine, CA USA; 71 Dent Neurologic Institute, Amherst, MA USA; 72 Ohio State University, Columbus, OH USA; 73 Albany Medical College, Albany, NY USA; 74 Hartford Hosp, Olin Neuropsychiatry Research Center, Hartford, CT USA; 75 Dartmouth Hitchcock Medical Center, Albany, NY USA; 76 Wake Forest University Health Sciences, Winston-Salem, NC USA; 77 Rhode Island Hospital, Rhode Island, USA; 78 Butler Hospital, Providence, RI USA; 79 Medical University South Carolina, Charleston, SC USA; 80 St. Joseph’s Health Care, Toronto, Canada; 81 Nathan Kline Institute, Orangeburg, SC USA; 82 University of Iowa College of Medicine, Iowa City, IA USA; 83 Cornell University, Ithaca, NY USA; 84 University of South Florida, USF Health Byrd Alzheimer’s Institute, Tampa, FL 33613 USA; 85 Memory Clinic, Ängelholm Hospital, Skåne, Sweden; 86 Department of Neurology, Skåne University Hospital, Skåne, Sweden; 87 Department of Neurosurgery, Skåne University Hospital, Skåne, Sweden; 88 MRI Unit, Lund University, Lund, Sweden; 89 Clinical Neurochemistry Laboratory, University of Gothenburg, Gothenburg, Sweden; 90 MRI Unit, Karolinska Institutet, Solna, Sweden; 91 PET UNIT, LUND UNIVERSITY, Lund, Sweden

**Keywords:** Network models, Alzheimer's disease

Correction to: *Nature Communications* 10.1038/s41467-020-15701-2, published online 26 May 2020.

The original version of the Supplementary information associated with this Article inadvertently omitted Supplementary Table S1. The HTML has been updated to include a corrected version of the Supplementary information.

## Supplementary information

Supplementary Information

