# Supplementary material for: Spread of pathological tau proteins through communicating neurons in human Alzheimer’s disease
Source: Nat Commun. 2020 May 26;11:2612. doi: 10.1038/s41467-020-15701-2 (PMC7251068; doi:10.1038/s41467-020-15701-2)
Supplement: Supplementary file 1 — Supplementary Information [file 41467_2020_15701_MOESM1_ESM.pdf]

Supplementary Information for Spread of pathological tau proteins through communicating neurons in human Alzheimer's disease

Jacob W. Vogel[a,*], Yasser Iturria-Medina[a], Olof T. Strandberg[b], Ruben Smith[b,c], Elizabeth Levitis[a], Alan C. Evans[a,**], Oskar Hansson[b,c,**], for the Alzheimer's Disease Neuroimaging Initiative, and the Swedish BioFinder Study

[a]*Montreal Neurological Institute, McGill University, Montréal, QC, Canada*
[b]*Clinical Memory Research Unit, Lund University, Lund, Sweden*
[c]*Memory Clinic, Skåne University Hospital, Lund, Sweden*

---

*Corresponding authors: jacob.vogel@mail.mcgill.ca, oskar.hansson@med.lu.se
**These authors jointly supervised this work

Data used in preparation of this article were obtained from the Alzheimer's Disease Neuroimaging Initiative (ADNI) database (adni.loni.usc.edu). As such, the investigators within the ADNI contributed to the design and implementation of ADNI and/or provided data but did not participate in analysis or writing of this report. A complete listing of ADNI investigators can be found at: http://adni.loni.usc.edu/wp-content/uploads/howtoapply/ADNIAcknowledgementList.pdf

	ADNI Tau				BioF			
	**CN**	**MCI**	**AD**	**Total**	**CN**	**MCI**	**AD**	**Total**
**n**	98	64	15	177	64	25	46	135
**Age (SD)**	70.5 (5.7)	70.9 (7.1)	74.5 (6.6)	71.0 (6.4)	74.4 (6.6)	70.7 (9.7)	71.2 (8.3)	72.6 (8.0)
**% Male**	56.10%	35.90%	40.00%	47.40%	53.10%	36.00%	41.90%	46.20%
**Education (SD)**	16.5 (2.4)	16.5 (3.0)	15.5 (2.7)	16.4 (2.7)	12.2 (3.7)	12.3 (3.3)	11.9 (3.9)	12.1 (3.7)
**APOE4 %**	36.30%	35.90%	60.00%	38.20%	48.40%	80.00%	64.10%	59.30%
**Aβ Positive %**	34.70%	50.00%	100.00%	45.80%	54.70%	100.00%	93.50%	75.20%

	ADNI rsfmri				ADNI DTI			
	**CN**	**MCI**	**AD**	**Total**	**CN**	**MCI**	**AD**	**Total**
**n**	60	96	33	189	54	108	42	204
**Age (SD)**	74.7 (6.7)	72.0 (7.3)	72.5 (7.2)	73.0 (7.2)	73.3 (5.7)	73.0 (7.2)	75.3 (8.5)	73.6 (7.2)
**% Male**	53.30%	51.00%	51.50%	51.90%	50.00%	35.70%	35.70%	39.70%
**Education (SD)**	16.8 (2.3)	16.1 (2.7)	15.6 (2.7)	16.24 (2.6)	16.3 (2.7)	15.9 (2.7)	15.35 (3.0)	15.9 (2.8)
**APOE4%**	38.20%	48.30%	77.40%	50.30%	33.30%	56.50%	63.40%	51.70%
**Aβ Positive %**	44.80%	62.70%	89.70%	61.80%	35.70%	61.00%	88.10%	59.90%

Supplementary Table. 1: Demographic information for each of the samples used in this study, stratified by clinical diagnosis.

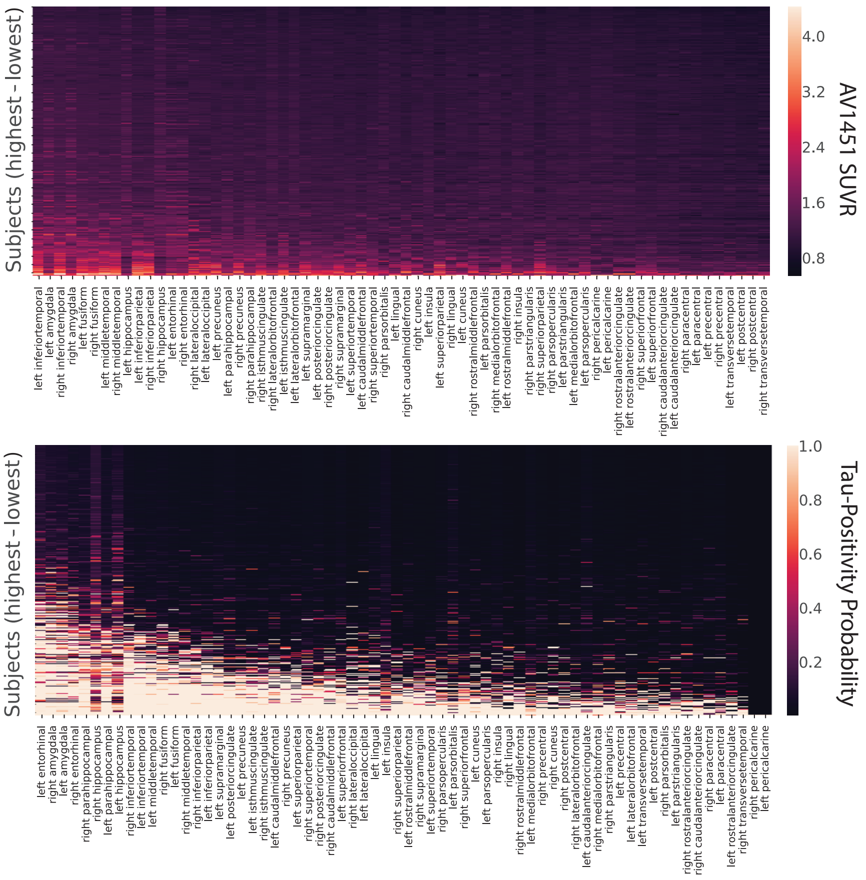

Supplementary Fig. 1: Tau-PET data before and after conversion to tau-positive probabilities. Each row is a subject sorted top-bottom by least to most overall tau. Each column is an ROI, sorted left to right by most to least overall tau. Warmer colors represent higher SUVR values (top) or tau-positive probabilities (bottom). Conversion to tau-positive probabilities creates a sparse distribution of values demonstrating a progression. The order of ROIs resembles those described in the autopsy literature.

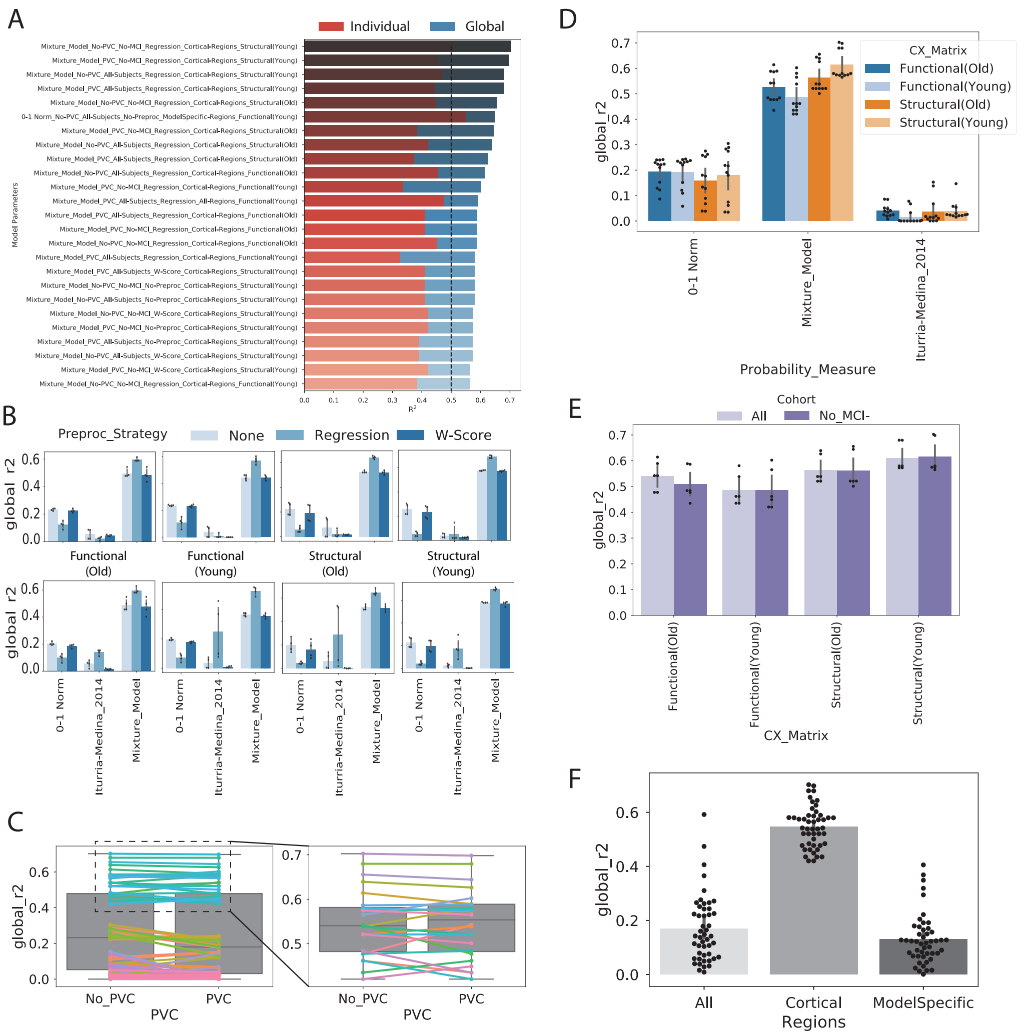

Supplementary Fig. 2: Impact of preprocessing decisions on model performance. (A) Parameters for the top 25 models. Blue bars represent global model fit, whereas red bars represent average within-subject fit. Models to the right of the black line explain more than half of the total variance in tau spatial pattern. (B) Influence of tau-PET input measure (i.e. tau probability; x-axis) and confound regression strategy (colors) on global model performance (y-axis) across all subjects (top) and Aβ- subjects only (bottom). Error bars represent variation in model fit depending on connectome, PVC strategy, and inclusion/exclusion of MCI- subjects. (C) Impact of PVC on model performance for all models (left) and models using tau-positive probabilities (right). Lines show change in model fit depending on PVC strategy. For boxplots, the center line = median, box = inner quartiles, whiskers = extent of data-distribution. (D) Impact of connectome choice (color) on variation in model performance (y-axis) across different tau input measures (x-axis). Error bars represent variation in model fit depending on PVC strategy, regression strategy and inclusion/exclusion of MCI- subjects. (E) Variation in model performance based on whether MCI- subjects were included or not, across different connectomes. Data are visualized only for models using the mixture modeling approach for tau probabilities. Error bars represent variation in model fit depending on PVC strategy and regression strategy (F) Model fit variation related to which regions were included. Only models using the mixture-modeling approach for tau probabilities are visualized, and error bars represent variation in model fit depending on PVC strategy, regression strategy, connectome and inclusion/exclusion of MCI- subjects. For all panels, error bars represent standard error of the mean.

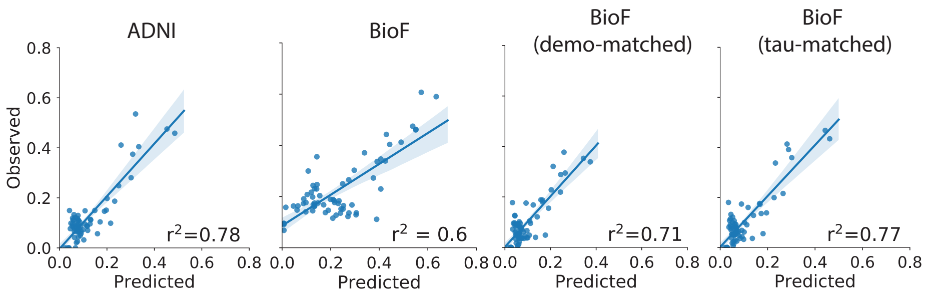

Supplementary Fig. 3: Global model fit for the best-fitting model averaged across (from left to right) ADNI subjects only, BioFINDER subjects only, a subselection of BioFINDER subjects matched to ADNI based on demographics, and a subselection of BioFINDER subjects matched to ADNI based on average cortical tau.

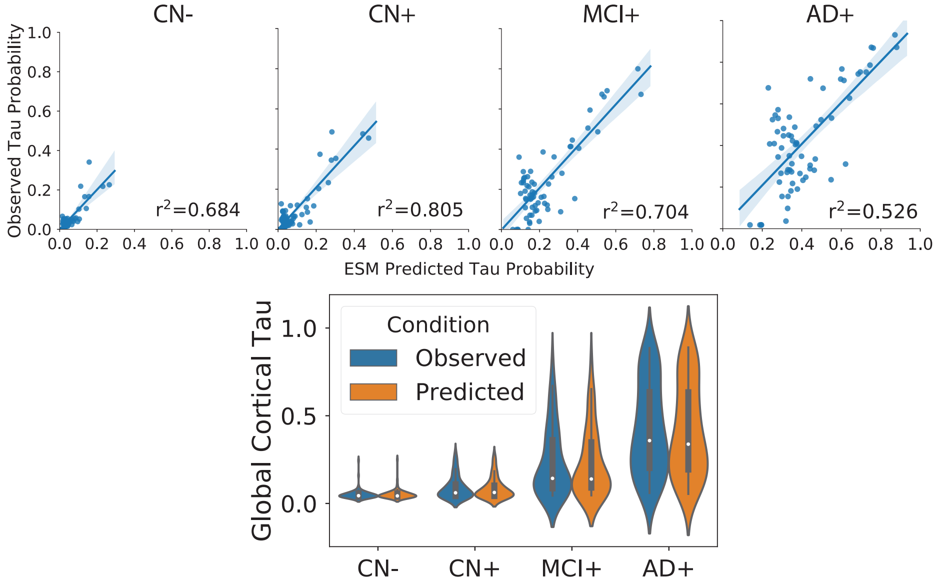

Supplementary Fig. 4: ESM performance across disease states. (Top) The ESM predicted the global tau pattern across disease states. (Bottom) Observed tau patterns increased with increased disease severity. By design, the predicted pattern also increases with disease progression, as each model is fit to the individual in terms of magnitude of tau (but not spatial pattern, which is determined by connectivity)

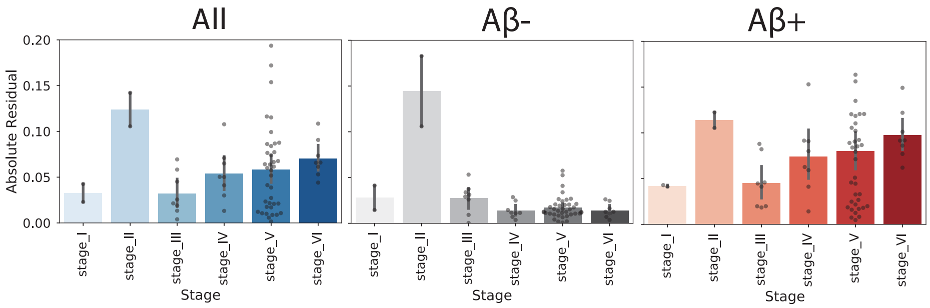

Supplementary Fig. 5: Model fit across disease progression. Regions were sorted into Braak Stage as in Main Text Fig 2. The average within-region model fit was calculated as the absolute difference (i.e. residual) between predicted and observed tau-probability in that region. This relationship is depicted across stages for the best-fitting model, across (left) all subjects, (middle) amyloid-negative subjects only and (right) amyloid-positive subjects only. In general, model fit became worse across as in regions belong to later Braak stages, and the hippocamus (Braak Stage II) was generally poorly fit. Error bars represent standard error of the mean.

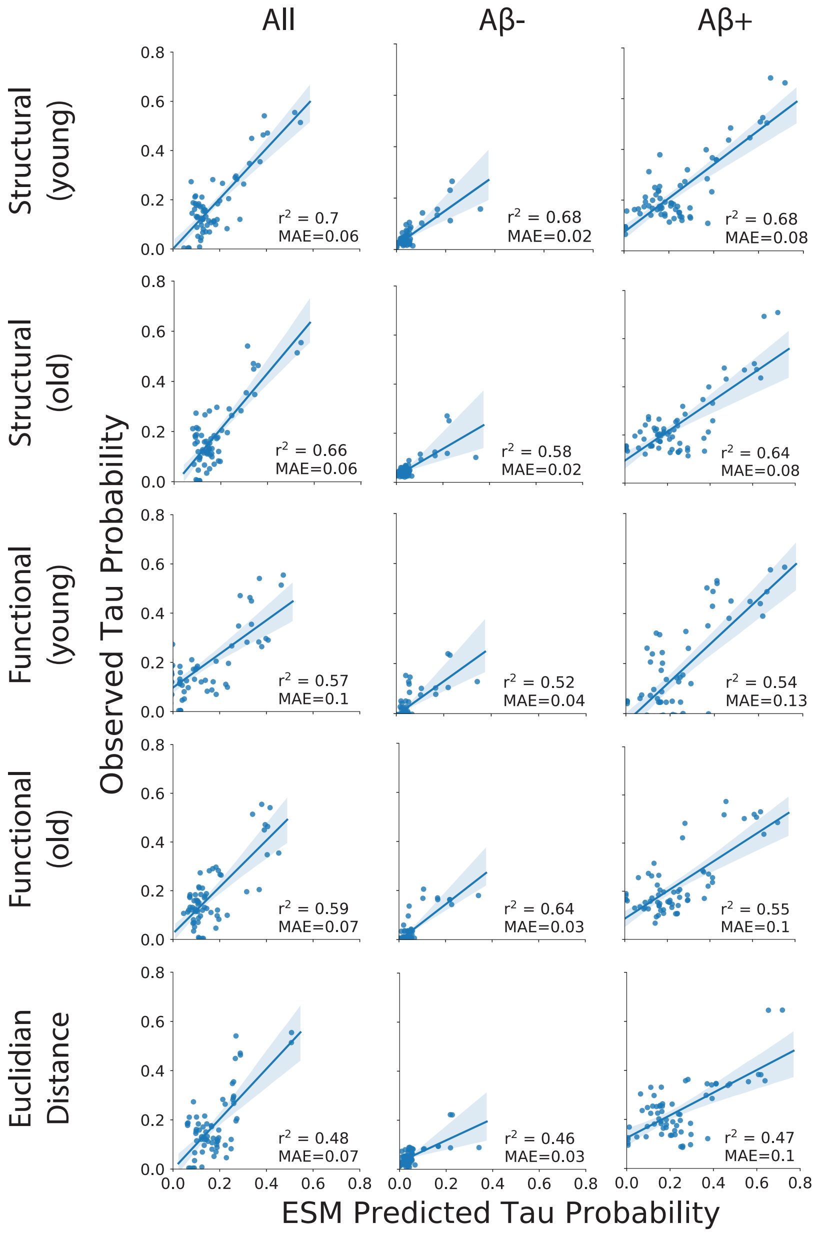

Supplementary Fig. 6: Performance of ESM in predicting spatial progression of tau. For each plot, each dot represents a region. The x-axis represents the mean simulated tau-positive probabilities across the population, while the y-axis represents the mean observed tau-positive probability. A value of (say) 0.3 for a given ROI would suggest that an average of 30% of all subjects included were predicted (X) or observed (Y) to have positive abnormal tau signal in that region. The results are shown for ESM fit over (from top to bottom) healthy young structural connectome (also selected as best-fitting); aging structural connectome; healthy young functional connectome; aging functional connectome; and a Eucidian distance matrix. B) Breakdown of ESM performance by amyloid status. The average performance of the four different models are shown separately for (left) all subjects, (center) Aβ- individuals and (right) Aβ+ individuals
