# Supplementary material for: Spread of pathological tau proteins through communicating neurons in human Alzheimer’s disease
Source: Nat Commun. 2020 May 26;11:2612. doi: 10.1038/s41467-020-15701-2 (PMC7251068; doi:10.1038/s41467-020-15701-2)
Supplement: Supplementary file 3 — Reporting Summary [file 41467_2020_15701_MOESM3_ESM.pdf]

nature research

Corresponding author(s): Oskar Hansson, Jacob Vogel

Last updated by author(s): March 1, 2020

Reporting Summary

Nature Research wishes to improve the reproducibility of the work that we publish. This form provides structure for consistency and transparency in reporting. For further information on Nature Research policies, see Authors & Referees and the Editorial Policy Checklist.

Statistics

For all statistical analyses, confirm that the following items are present in the figure legend, table legend, main text, or Methods section.

n/a	Confirmed	
☐	☒	The exact sample size (*n*) for each experimental group/condition, given as a discrete number and unit of measurement
☐	☒	A statement on whether measurements were taken from distinct samples or whether the same sample was measured repeatedly
☐	☒	The statistical test(s) used AND whether they are one- or two-sided<br>*Only common tests should be described solely by name; describe more complex techniques in the Methods section.*
☐	☒	A description of all covariates tested
☐	☒	A description of any assumptions or corrections, such as tests of normality and adjustment for multiple comparisons
☐	☒	A full description of the statistical parameters including central tendency (e.g. means) or other basic estimates (e.g. regression coefficient) AND variation (e.g. standard deviation) or associated estimates of uncertainty (e.g. confidence intervals)
☐	☒	For null hypothesis testing, the test statistic (e.g. *F*, *t*, *r*) with confidence intervals, effect sizes, degrees of freedom and *P* value noted<br>*Give P values as exact values whenever suitable.*
☒	☐	For Bayesian analysis, information on the choice of priors and Markov chain Monte Carlo settings
☒	☐	For hierarchical and complex designs, identification of the appropriate level for tests and full reporting of outcomes
☐	☒	Estimates of effect sizes (e.g. Cohen's *d*, Pearson's *r*), indicating how they were calculated

Our web collection on statistics for biologists contains articles on many of the points above.

Software and code

Policy information about availability of computer code

Data collection	No software was used for data collection; data preprocessing was performed using an in-house pipeline including ANTs v 2.1, AFNI v 20.0.18, and Freesurfer v 6.0
Data analysis	The main analysis was computed using the Epidemic Spreading Model, one item in a suite of analyses offered by an yet-to-be published software package. However, if the software is not published in time, we will archive the code used for these analyses on GitHub. All additional statistical analysis was completed using Python 3.6. The scripts used for analysis can be found here: https://github.com/illdopejake/data_driven_pathology/tree/master/esm .

For manuscripts utilizing custom algorithms or software that are central to the research but not yet described in published literature, software must be made available to editors/reviewers. We strongly encourage code deposition in a community repository (e.g. GitHub). See the Nature Research guidelines for submitting code & software for further information.

Data

Policy information about availability of data

All manuscripts must include a data availability statement. This statement should provide the following information, where applicable:

- Accession codes, unique identifiers, or web links for publicly available datasets
- A list of figures that have associated raw data
- A description of any restrictions on data availability

Tau-PET data stemmed from two sources: the Alzheimer's Neuroimaging Disease Initiative (ADNI) and the Swedish BioFINDER study. ADNI data is available to the public and can be downloaded from adni.loni.usc.edu after signing up and completing a data-use agreement. ADNI Diffusion MRI data were also used for tractographic analysis and resting-state fMRI data were used for connectivity. Data from the Swedish BioFINDER study is not public, but data can be made available to independent researchers upon request. In addition, resting-state functional imaging data stemmed from the Cobre dataset, a public access dataset that was downloaded directly through the nilearn software. See here for details: http://fcon_1000.projects.nitrc.org/indi/retro/cobre.html. The public-access CMU60 DTI dataset was additionally used to for tractographic analyses. See here for details: http://brain.labsolver.org/diffusion-mri-templates/cmu-60-template

Field-specific reporting

Please select the one below that is the best fit for your research. If you are not sure, read the appropriate sections before making your selection.

☒ Life sciences ☐ Behavioural & social sciences ☐ Ecological, evolutionary & environmental sciences

For a reference copy of the document with all sections, see nature.com/documents/nr-reporting-summary-flat.pdf

Life sciences study design

All studies must disclose on these points even when the disclosure is negative.

Sample size

312 individuals spanning the Alzheimer's Disease spectrum were included in this study from two different datasets. All individuals available that met inclusion criteria (at least one Tau-PET scan and one amyloid-biomaker (PET or CSF); diagnosis of cognitively normal, mild cognitive impairment, Alzheimer's disease with amyloid biomarkers) were included.

Data exclusions

Tau-PET scans for patients with other dementias were available, but not included. Similarly, patients with a clinical diagnosis of Alzheimer's disease but without biomarker evidence for cortical beta-amyloid were excluded. We excluded these groups because we were interested in tau propagation in Alzheimer's disease, and did not wish to confound our results by introducing other forms of dementia.

Replication

We successfully replicated our results using different connectivity data from two different samples using two different imaging modalities: resting-state functional connectivity and diffusion tractography. Additionally, we showed that our results are reproducible using several different processing pipelines, but that results were more dependent on certain processing decisions (i.e. the type of data normalization and the specific regions included).

Randomization

All data available fitting inclusion criteria was used for analysis. Subjects were separated into groups based on amyloid status, but this grouping was based on biomarker evidence for amyloid and therefore was not random. However, in the Swedish BioFINDER cohort only, ~50% and ~90% of cognitively normal controls and MCI subjects scanned, respectively, had biomarker evidence for amyloid by design.

This is probably not relevant, but we showed that regression of confounds for sex and choroid plexus binding improved our model fit.

Blinding

Technicians processing PET data were blind to the disease status and demographic information of individuals.

Reporting for specific materials, systems and methods

We require information from authors about some types of materials, experimental systems and methods used in many studies. Here, indicate whether each material, system or method listed is relevant to your study. If you are not sure if a list item applies to your research, read the appropriate section before selecting a response.

Materials & experimental systems

n/a	Involved in the study	
☒	☐	Antibodies
☒	☐	Eukaryotic cell lines
☒	☐	Palaeontology
☒	☐	Animals and other organisms
☐	☒	Human research participants
☒	☐	Clinical data

Methods

n/a	Involved in the study	
☒	☐	ChIP-seq
☒	☐	Flow cytometry
☐	☒	MRI-based neuroimaging

Human research participants

Policy information about studies involving human research participants

Population characteristics

This information can is detailed Table 1 of the manuscript. The samples was composed of 162 cognitively unimpaired individuals, 89 individuals with mild cognitive impairment, and 61 patients with Alzheimer's disease. The mean age of the sample was around 72, with about 53% of the sample composed of women, and the average years of education about 15. 52% of the sample carried an APOE4 allele.

Recruitment

Recruitment details for the Swedish BioFINDER study have been published online: https://clinicaltrials.gov/ct2/show/NCT01208675. In addition, in the BioFINDER cohort only, ~50% and ~90% of cognitively normal controls and MCI subjects scanned, respectively, had biomarker evidence for amyloid by design. This choice was made to increase likelihood of recruiting individuals with measurable tau signal, given the high costs of tau-PET scans. While this means the BioFINDER data may not be representative of a population, it would be representative of a clinical population, where tau scans would likely only be ordered in the event of other evidence of Alzheimer's disease, or a need to exclude it.

Ethics oversight

BioFINDER: PET imaging for the study was approved by the Swedish Medicines and Products Agency and the local Radiation

Ethics oversight	Safety Committee at Skåne University Hospital, Sweden. All participants provided written informed consent according to the Declaration of Helsinki, and ethical approval was given by the Ethics Committee of Lund University, Lund, Sweden. Ethics information for ADNI (adni.loni.usc.edu) and Cobre (http://fcon_1000.projects.nitrc.org/indi/retro/cobre.html) can be found through their respective weblinks.

Note that full information on the approval of the study protocol must also be provided in the manuscript.

Magnetic resonance imaging

Experimental design

Design type	Resting-state
Design specifications	5-minute resting state scan
Behavioral performance measures	n/a -- resting state

Acquisition

Imaging type(s)	functional; diffusion
Field strength	3 Tesla
Sequence & imaging parameters	fMRI: Rest data was collected with single-shot full k-space echo-planar imaging (EPI) with ramp sampling correction using the intercomissural line (AC-PC) as a reference (TR: 2 s, TE: 29 ms, matrix size: 64x64, 32 slices, voxel size: 3x3x4 mm3).
Area of acquisition	Whole-brain

Diffusion MRI ☒ Used ☐ Not used

Parameters	35 cm field of view, 128×128 acquired matrix, reconstructed to a 256×256 matrix; voxel size: 1.35×1.35×2.7mm ; scan time = 9 min; 41 diffusion-weighted directions at b-value = 1000 s/mm2 and 5 T2-weighted images (b-value = 0 s/mm2)

Preprocessing

Preprocessing software	Freesurfer, AFNI, ANTs
Normalization	PET data were coregistered to native space T1s using AFNIs 3D VolReg. No spatial normalization took place -- all ROI-level data was extracted from native space.<br><br>The fMRI derivates involved spatial normalization as a preprocessing step, though this step was not performed by us. From https://figshare.com/articles/Cobre_for_machine_learning/1450804:<br>The median volume of one selected fMRI run for each subject was coregistered with a T1 individual scan using Minctracc (Collins and Evans, 1998), which was itself non-linearly transformed to the Montreal Neurological Institute (MNI) template (Fonov et al., 2011) using the CIVET pipeline (Ad-Dabbagh et al., 2006). The MNI symmetric template was generated from the ICBM152 sample of 152 young adults, after 40 iterations of non-linear coregistration. The rigid-body transform, fMRI-to-T1 transform and T1-to-stereotaxic transform were all combined, and the functional volumes were resampled in the MNI space at a 3 mm isotropic resolution.
Normalization template	All comparisons were performed in native space.
Noise and artifact removal	Each fMRI dataset was corrected for inter-slice difference in acquisition time and the parameters of a rigid-body motion were estimated for each time frame. Rigid-body motion was estimated within as well as between runs, using the median volume of the first run as a target. The following nuisance parameters were regressed out from the time series at each voxel: slow time drifts (basis of discrete cosines with a 0.01 Hz high-pass cut-off), average signals in conservative masks of the white matter and the lateral ventricles as well as the first principal components (95% energy) of the six rigid-body motion parameters and their squares (Giove et al., 2009).
Volume censoring	A scrubbing procedure was used to remove volumes of >0.5 frame displacement.

Statistical modeling & inference

Model type and settings	The Epidemic Spreading model involves simulating the diffusion of a signal through a system of connected nodes. The simulation was based on human functional connectivity data and human tau-PET data. The model is fit within-subject, and multiple simulations are performed in order to fit a set of three parameters. These parameters define magnitude but not direction of spread. We compare the result of our simulations to the observed pattern of tau-PET spread using simple linear model evaluation metrics (i.e. R-square).
Effect(s) tested	We test the whether we can recreate the observed pattern of tau-PET signal in the brain by allowing an artificial signal to diffuse from a pathologically defined epicenter through a system of macroscale human brain connections.

Specify type of analysis: ☐ Whole brain ☒ ROI-based ☐ Both

Anatomical location(s)	The Desikan-Killiainy atlas was used -- this atlas was created using automated segmentation of anatomical T1 images.
Statistic type for inference (See Eklund et al. 2016)	We do not perform voxelwise analysis in our study. Our principal analyses involve fitting a simulation to explain tau spread, and most of the testing involves evaluating model fit across the whole brain.
Correction	Only one analysis involves multiple comparisons (Figure 7F), and here we use FDR correction for the number of regions of interest.

Models & analysis

n/a	Involved in the study
☐	☒ Functional and/or effective connectivity
☒	☐ Graph analysis
☒	☐ Multivariate modeling or predictive analysis

Functional and/or effective connectivity	Pearson correlation, thresholded and normalized to a 0-1 scale
